# Supplementary material for: A low-dimensional approximation of optimal confidence
Source: PLoS Comput Biol. 2024 Jul 24;20(7):e1012273. doi: 10.1371/journal.pcbi.1012273 (PMC11299811; doi:10.1371/journal.pcbi.1012273)
Supplement: S1 Text — (PDF) [file pcbi.1012273.s001.pdf]

S1 Text. Explicit derivation from DDM assumptions to our formulation of confidence.

We here write out in detail the derivation of the model. We define confidence to be the probability of being correct given the available evidence (e) and elapsed time (t). Under DDM assumptions, The probability density of drift rate (v) is normally distributed with mean e/t and with variance σ^2/t [1]. To find the correct expression for the probability correct, We must split this up for the two possible responses that can be given ($x = 1, -1$). We find that when $x = 1$, the DDM holds that $Pr(correct \mid e, t, x = 1)$ equals the probability that v is larger than zero:

$$\begin{aligned} & Pr(v > 0 \mid e, t, x = 1) \\ &= \int_0^{+\infty} \phi\left(\frac{v - e/t}{\sigma/\sqrt{t}}\right) dv \\ &= \int_{-e/(\sigma\sqrt{t})}^{+\infty} \phi(v) dv \\ &= 1 - \Phi\left(\frac{-e}{\sigma\sqrt{t}}\right) \\ &= \Phi\left(\frac{e}{\sigma\sqrt{t}}\right) \\ &\approx Logist\left(\frac{e}{\sigma\sqrt{t}}\right) \end{aligned} \tag{1}$$

where ϕ is the probability density function of the normal distribution and Φ its cumulative distribution function. In the case where $x = -1$, we need to find $Pr(correct \mid e, t, x = -1)$ and this is the probability that v is smaller than zero:

$$\begin{aligned} & Pr(v < 0 \mid e, t, x = -1) \\ &= \int_{-\infty}^{0} \phi\left(\frac{v - e/t}{\sigma/\sqrt{t}}\right) dv \\ &= \int_{-\infty}^{-e/(\sigma\sqrt{t})} \phi(v) dv \\ &= \Phi(-e) \\ &\approx Logist\left(\frac{-e}{\sigma\sqrt{t}}\right) \end{aligned} \tag{2}$$

Combining these two results, we obtain our equation for confidence.

References

[1] R. Moreno-Bote. Decision Confidence and Uncertainty in Diffusion Models with Partially Correlated Neuronal Integrators. *Neural Computation*, 22(7):1786–1811, July 2010.
