# Supplementary material for: A low-dimensional approximation of optimal confidence
Source: PLoS Comput Biol. 2024 Jul 24;20(7):e1012273. doi: 10.1371/journal.pcbi.1012273 (PMC11299811; doi:10.1371/journal.pcbi.1012273)
Supplement: S1 Fig — (PDF) [file pcbi.1012273.s002.pdf]

S1 Fig. Confidence decreases with RT in both behavioral data and LDC model predictions. We plot below the relationship between confidence and RT for Experiment 1 and 2 (both empirical data and model predictions). Since there were very few trials with longer RTs, we grouped all RT > 1.5 s trials in the same bin (5.6% of all trials in Experiment 1, 9.9% in Experiment 2A and 11.3% in Experiment 2B). As predicted by both the LDC model and the optimal model for confidence (i.e. the Bayesian readout described in Eq. 1 of the main manuscript), and already identified in previous works (1,2), we observed a decrease in confidence with longer RTs in all experiments (all mean Pearson correlation coefficients across participants *r*s < -.12, all *p*s < .001).

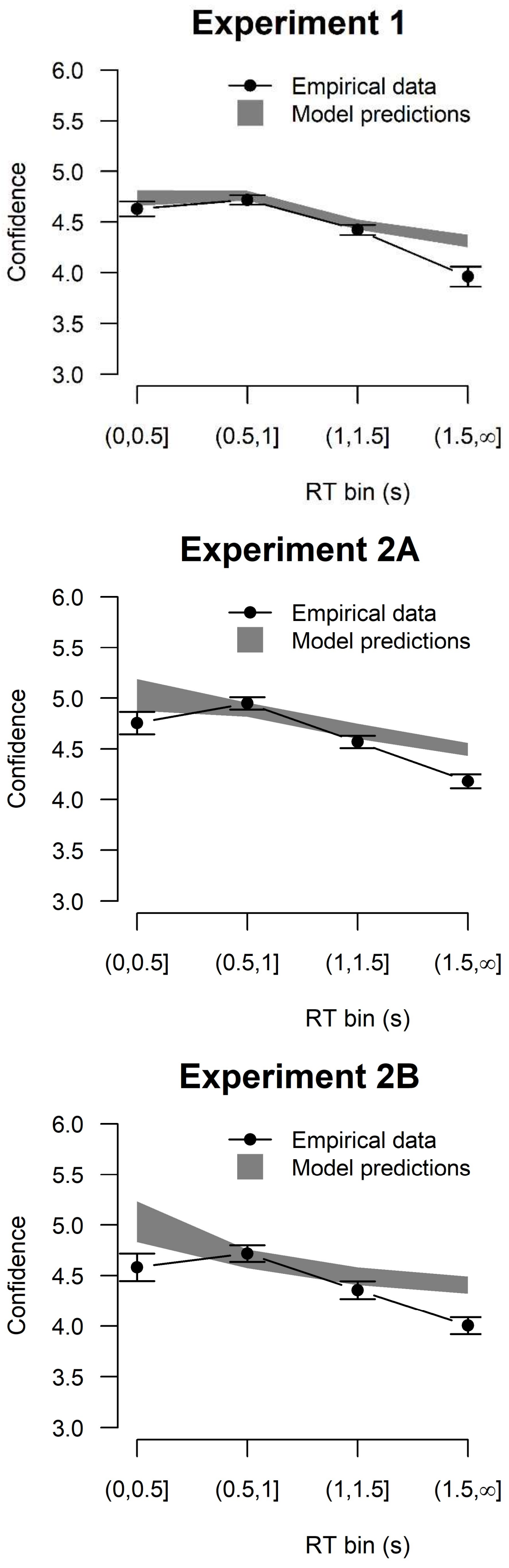
Experiment 1
Experiment 2A
Experiment 2B
Empirical data
Model predictions
Confidence
6.0
5.5
5.0
4.5
4.0
3.5
3.0
(0,0.5]
(0.5,1]
(1,1.5]
(1.5,∞]
RT bin (s)

References

1. Desender K, Donner TH, Verguts T. Dynamic expressions of confidence within an evidence accumulation framework. Cognition. 2021 Feb 1;207:104522.

2. Kiani R, Corthell L, Shadlen MN. Choice Certainty Is Informed by Both Evidence and Decision Time. Neuron. 2014 Dec 17;84(6):1329–42.
